# Evaluation of the Fecal Proteome in Healthy and Diseased Cheetahs (*Acinonyx jubatus*) Suffering from Gastrointestinal Disorders

**DOI:** 10.3390/ani12182392

**Published:** 2022-09-13

**Authors:** Sara Mangiaterra, Silvia Vincenzetti, Giacomo Rossi, Andrea Marchegiani, Alessandra Gavazza, Thierry Petit, Gianni Sagratini, Massimo Ricciutelli, Matteo Cerquetella

**Affiliations:** 1 School of Biosciences and Veterinary Medicine, University of Camerino, Via Circonvallazione 93/95, 62024 Matelica, MC, Italy; 2 Zoo La Palmyre, 17570 Les Mathes, France; 3 School of Pharmacy, University of Camerino, Via Sant’Agostino, 1, 32032 Camerino, MC, Italy

**Keywords:** *Acinonyx jubatus*, cheetah, fecal proteomics, gastrointestinal disorder, *Helicobacter* spp.

## Abstract

**Simple Summary:**

The cheetah (genus *Acinonyx*), which is considered vulnerable by the IUCN Red List, suffers quite frequently from chronic gastrointestinal (GI) diseases, often associated with *Helicobacter* spp., with a significant mortality, especially in captive animals. The study of the fecal proteome aims to investigate possible new markers of disease that could be useful in diagnosing and/or monitoring, when performed in feces, gastrointestinal disorders. Considering the invasiveness of some diagnostic investigations (e.g., GI endoscopy) in general and the difficulties of carrying out even simple blood sampling in animals such as the cheetah, the possibility of deriving clinical-diagnostic information from fecal samples is extremely interesting. Herein, a fecal proteomic evaluation from healthy and diseased cheetahs suffering from GI disorders is performed.

**Abstract:**

Fecal proteomics allows for the identification of proteins and peptides present in stools and is useful in finding possible new biomarkers for diagnosing and/or monitoring gastrointestinal (GI) disorders. In the present study, we investigated the fecal proteome in healthy and diseased cheetahs (*Acinonyx jubatus*). Captive individuals of this species frequently show gastrointestinal disorders characterized by recurrent episodes of diarrhea, rare episodes of vomiting and weight loss, associated with *Helicobacter* spp. infection. Fecal proteomic evaluation has been performed by two-dimensional electrophoresis followed by liquid chromatography-tandem mass spectrometry. In healthy cheetahs, the results showed the presence of the following proteins: collagen alpha-1 (II) chain, transthyretin, IgG Fc-binding protein, titin, dystonin, isopentenyl-diphosphate Delta-isomerase 1, sodium/potassium-transporting ATPase subunit alpha-1 and protein disulfide-isomerase A6. The presence of albumin isoforms was found only in diseased cheetahs. The present paper reports the study of the fecal proteome in the cheetah, evidences some differences between healthy and diseased patients and confirms, once again, the potential of fecal proteomics for the study of the GI environment, with promising developments regarding the identification of new diagnostic/monitoring markers.

## 1. Introduction

Since the 1900s, the cheetah has achieved a unique taxonomic status as the only species of the genus *Acinonyx* and has been classified as vulnerable by the IUCN Red List [1]. Today, the known adult population is of about 7000 animals, representing a decline of at least 30% in the last 20 years [1]. The decline in the cheetah’s population has increased the focus on the conservation of this species and on the study of chronic diseases. Gastrointestinal (GI) diseases include a high number of recorded cases associated with *Helicobacter* organisms, with significant mortality in cheetahs, especially in captive animals [2]. Inconstant clinical signs including vomiting, diarrhea and weight loss are described in cheetahs affected by chronic gastro-enteric disorders. *Helicobacter* species identified in cheetahs affected by gastritis are *Helicobacter acinonychis* (or *H. acinonyx),* associated with lymphoplasmacytic gastric infiltration, and *H. heilmannii*, which seems to be less frequently associated with gastric diseases [3]. To date, the diagnosis is made through the histological examination of gastrointestinal biopsies. This procedure represents an invasive practice that requires the anesthesia of the animal. The term “proteome” describes the set of proteins encoded by the genome [4]. The study of the proteome is called “proteomics”, including all protein isoforms and modifications [5]. The identification of proteins and peptides present in stools could be of great interest, since they may be related to different bowel-related diseases. In this regard, fecal samples can be considered an accessible, alternative biological sample to investigate a range of diseases of the gastrointestinal tract [6]. In this study, the results obtained in patients suffering from gastrointestinal disorders were compared to those of healthy controls. This work aims to better understand the cheetah’s GI environment, the pathophysiology of related diseases and the discovery of new diagnostic/monitoring markers.

## 2. Materials and Methods

### 2.1. Patients and Controls

In the present study, naturally voided fecal samples from 11 cheetahs were included: 7 with a clinical history of recurrent gastrointestinal disorders associated with *Helicobacter* spp. infection (diseased cheetahs—DC), and 4 healthy subjects (healthy cheetahs—HC). In both groups, other pathologies were excluded by complete blood tests and serum biochemical examinations that resulted within the reference ranges (globulins; α1 globulins, α2 globulins, β globulins, γ globulins; total proteins (TP); albumin (Alb); alkaline phosphatase (ALP); glutamic pyruvic transaminase (GPT); alanine transaminase (ALT); gamma-glutamyl transferase (GGT); aspartate transferase (AST); glucose (GLU); lipase (LP); sodium (Na); potassium (K); calcium (Ca); chloride (Cl); phosphorus (P); magnesium (Mg); cholesterol (Col); triglycerides (Tri); creatinine (Crea); BUN, symmetric dimethylarginine (SDMA)); all subjects were also negatively tested for FIV-FeLV. The HC group was made up of two males and two females, between 5 and 11 years of age, with no clinical history of gastrointestinal disease or any other pathology, and they tested negative for *Helicobacter* spp. in fecal samples by rt-PCR. The DC group was made up of four males and three females, between 7 and 11 years of age, with a clinical history of GI disorders characterized by recurrent episodes of diarrhea, rare episodes of vomiting and weight loss. In the DC group, all cheetahs tested positive for *Helicobacter* spp. in fecal samples by rt-PCR analysis. All of the animals included in the study did not undergo dietary changes or medical treatments (in the two months prior to the study); parasitic diseases were also excluded by the fecal examination performed in the zoos of origin. The cheetahs’ blood sampling related to the data reported herein was performed for routine programmed screening/monitoring evaluations, which was in the interest of the patient. The University of Camerino’s Convention on International Trade in Endangered Species of Wild Fauna and Flora (CITES) Scientific Institution Code: IT034.

### 2.2. Sample Preparation and Two-Dimensional Electrophoresis (2DE)

The protocol for fecal protein extraction and subsequent two-dimensional electrophoresis (2DE) has been described in previous works [7,8,9]. Briefly, two grams of feces from each healthy (HC) and diseased (DC) animal were weighed and pooled together, amounting to a total of 22.0 g of feces from both groups. To each pooled group sample, 60 mL of phosphate-buffered saline (PBS) containing a 1:100 diluted protease inhibitor cocktail (Sigma-Aldrich, Saint Louis, MO, USA) was added. Each group sample was separately subjected to agitation through a magnetic stirrer for 60 min at 4 °C. Subsequently, the samples were centrifuged at 10,000× *g* for 20 min at 4 °C, and supernatants from each group were recovered and separately filtered three times with a paper filter. The resulting filtrates were subjected to two filtering steps with 0.45 mm and 0.22 mm filters (Whatman, Maidstone, UK). The final steps foresee protein precipitation using 90% of ammonium sulphate (Sigma-Aldrich, Saint Louis, MO, USA), followed by centrifugation at 10,000× *g* for 30 min at 4 °C. Each protein pellet (from both HC and DC) was resuspended in 500 mL PBS, and the total protein content was determined by the Bradford method [10].

Before 2DE, one milligram of each group sample was separately treated with a 2-D Clean-Up Kit (GE-Healthcare Life Sciences, Uppsala, Sweden) to remove contaminants, and the final protein pellets were resuspended in a 350 mL rehydration buffer (8 M urea; 2% 3-[(3-Cholamidopropyl) dimethylammonio]-1-propanesulfonate, CHAPS; 65 mM dithiothreitol, DTT; 0.001% bromophenol blue; 0.5%IPG buffer, pH range 3–10). The first dimension (isoelectric focusing) was performed using a pre-cast ImmobilineDryStrip, (IPG-strip, length 18 cm, linear pH gradient 3–10). The apparatus was a IPGphor-isoelectric focusing cell (GEHealthcare). The focusing was performed at 20 °C as follows: rehydration for 12 h at 20 °C without voltage; 500 V for 1 h; 1000 V for 1 h; 8000 V for 4 h. The current limit per IPG-strip was 50 mA. After the first dimension, the strips were incubated for 15 min at room temperature in the equilibration buffer (50 mM Tris/HCl, pH 8.8; 6 M urea; 30% glycerol; 2% SDS; 65 mM DTT, few grains of bromophenol blue) and loaded on a 13% SDS-PAGE using a Protean II apparatus (Bio-Rad, Hercules, CA, USA). After electrophoresis, the gels were stained with 0.1% Coomassie Brilliant Blue R250 and then de-stained until the protein spots became evident and the gel background became transparent.

The PDquest software (Version 7.1.1; Bio-Rad Laboratories) was used to analyze the gel images (scanned at 600 dpi resolution) and the isoelectric point (pI); the molecular mass and the normalized quantity of each protein spot were calculated according to the protocol provided by the software manufacturer. In particular, the pIs of the single spots were determined using a linear 3–10 distribution, and molecular mass determinations were based on the markers (Prestained Protein Sharpmass VII; range 6.5–270 kDa; Euroclone, Pero, MI, Italian). For each group (HC and DC), 2DE analyses were performed in triplicate. The quantitative data of the protein level in both the HC and DC groups are presented as means ± SE.

### 2.3. Liquid Chromatography-Tandem Mass Spectrometry (LC-MS/MS)

Target protein spots were manually excised from the gel, cut into small pieces and subjected to the protein in-gel digestion procedure described by Shevchenko and co-workers [11]. Briefly, the excised spots were first destained with 100 mL of 100 mM ammonium bicarbonate/acetonitrile (1:1, *v*/*v*) and were subsequently treated with neat acetonitrile until the pieces became white. After acetonitrile removal, the gel pieces were incubated with trypsin (13 mg/mL trypsin in 10 mM ammonium bicarbonate containing 10% (*v*/*v*) acetonitrile) for 30 min on ice to allow the trypsin to diffuse into the gel. Thereafter, the pieces were incubated overnight at 37 °C. The resulting peptides were extracted from the gel by adding 100 mL of 5% formic acid (*v*/*v*): acetonitrile (1:2) to the gel pieces and incubating the mixture for 15 min at 37 °C in a shaker. The extract was recovered and dried in a vacuum concentrator at room temperature. A piece of blank gel without spots and a piece of lysozyme from the molecular mass markers were submitted to the same procedure and used as negative and positive controls, respectively. The resulting tryptic peptides were resuspended in 100 mL of 0.1% (*v*/*v*) trifluoroacetic acid and loaded into a reversed phase chromatograph (C18 Gemini-NX, 5 m particle size, 110 Å pore size, 250 × 4.6 mm, Phenomenex, Torrance, CA, USA) connected to an Agilent Technologies 1100 Series (Agilent Technologies, Santa Clara, CA, USA). The chromatographic conditions were as follows: flow rate 1 mL/min, 45 min linear gradient from 90:10% A:B to 10:90% A:B (buffer A is made of 0.1% formic acid in water, and buffer B is made of 0.1% formic acid in acetonitrile), column temperature 40 °C. The column effluent was analyzed by MS using an electrospray ion trap mass spectrometer (Agilent Technologies LC/MSD Trap SL) operating in positive ion mode over the mass range of 300–2200 amu (atomic mass units). The MS spray voltage was 3.5 kV, and the capillary temperature was maintained at 300 °C. The obtained MS spectra were extracted and analyzed by the MASCOT software (http://www.matrixscience.com/, accessed on 10 March 2022) with the following search parameters: database, SwissProt; Taxonomy, *Mammalia*; Enzyme, trypsin; peptide tolerance, 1.2 Da; MS/MS tolerance, 0.6 Da; and the allowance of one missed cleavage.

## 3. Results

**Figure 1:**
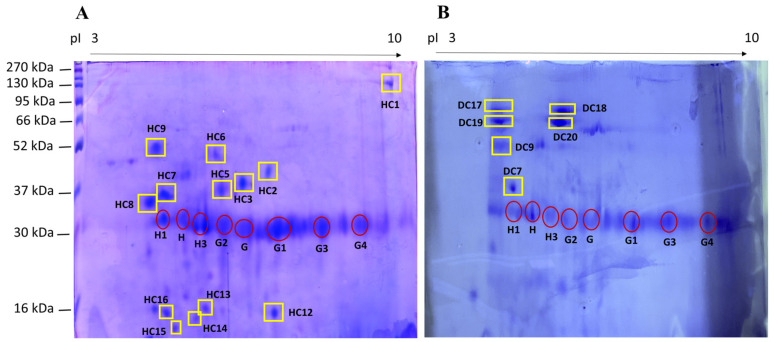
2DE proteomic map of feces from (**A**) healthy cheetahs (HC); (**B**) diseased cheetahs (DC). The proteins were separated on an immobilized pH 3–10 linear gradient strip and subsequently subjected to 13% SDS-PAGE. The standards were: Prestained Protein Sharpmass VII, range 6.5–270 kDa (Euroclone). Highlighted with a yellow square are the spots identified in this work by mass spectrometry. The spots identified by comparing the gels obtained from HC and DC with those already present in the literature [7,8,9] are highlighted with red circles.

The protein expression profiles of the fecal samples of HC and DC were examined by 2DE in the pH range of 3–10. The resulting gels are shown in Figure 1.

First, 13 spots were selected in the HC group, many of which were not present in the DC group. On the other hand, in the latter group, there were four spots that were not present in the HC group (from DC17 to DC20; Figure 1). In the HC group, 12 spots (out of 13—not the HC7) were identified with LC-MS/MS followed by MASCOT analysis (for a total of 8 proteins, considering duplicates), whereas, in the DC group, 3 spots (out of 6—not DC7, DC18 and DC19) were identified (for a total of 2 proteins, considering duplicates); the results are shown in Table 1.

In addition to the aforementioned spots, the proteomic maps of the healthy and diseased cheetahs were compared with the proteomic maps from healthy and diseased dogs’ feces already present in the literature [7,9]. The spots named H, H1, H3, G, G1, G2, G3 and G4 (evidenced by red circles in Figure 1) were consistently present in all of the stool samples examined and were assumed to correspond, as previously reported [7,9], to: Chymotrypsin-C-like (spot H), Elastase-3B (spot H1), Immunoglobulin kappa light chain (spot H3), Immunoglobulin λ − 1 light chain (spots G and G1) and Immunoglobulin λ-light chain VLJ (spots G2, G3 and G4).

## 4. Discussion

The present study represents the first fecal proteome investigation in cheetahs with GI disorders compared with healthy cheetahs.

In the diseased cheetahs, the spots DC17 and DC20 were identified as albumin isoforms, the spots DC18 and DC19 were not identified by mass spectrometry but could likely also correspond to albumin isoforms since they showed, after the PDQuest software analysis, a molecular weight and a pI corresponding to albumin and very close to the values found for the spots DC17 and DC20. Albumin (and relative isoforms) was previously found in both healthy dogs and cats [7] and was interpreted as the result of the physiological mucosal cellular turnover. In the case of DC, considering that albumin was found only in this group and not in the healthy one, it is possible to hypothesize that its presence may be due to the damage associated with GI disturbances, as is also assumed for the dogs suffering from intestinal lymphangiectasia [9].

Interpreting the findings from healthy cheetahs, the spot HC1 was assigned to the Collagen alpha-1 (II) chain, which is specific for cartilaginous tissues. Type II collagen is fundamental for the normal embryonic development of the skeleton and for the ability of cartilage to resist compressive forces. Spots HC2, HC3 and HC5 correspond to the transthyretin isoform, a protein whose main role is to transport, in plasma, retinol and thyroid hormones and is synthetized in the liver, brain and pancreas [9,12]. This protein is a 56-kDa protein composed of four identical subunits, and it contains a free cysteine thiol group residue prone to oxidation that gives rise to transthyretin isoforms. The modifications of this cysteine residue may reflect oxidative stress and are potentially related to transthyretin amyloidosis and Alzheimer’s disease [13,14]. Indeed, it is interesting that the amyloidogenic mutations of such protein have been associated with amyloids deposits (transthyretin amyloidosis), possibly correlated with the progressive damage of the autonomic nervous system, which may be in turn be associated with reduced GI motility [9,15]. Transthyretin is also considered a negative acute phase protein, as it commonly decreases in human neurological disorders, notwithstanding the fact that it was found to be upregulated, in a corticosteroid-related way, in dogs suffering from cervical spondylomyelopathy [16] but was also found to be decreased in dogs with pyometra [17]. This protein was also reported in a study conducted in 2017 involving the protein profiles of the fecal material of female cheetahs [18] and pregnant polar bears [19]. Moreover, in recent studies on the fecal proteome, it has been found to be more abundant in dogs presenting with canine chronic enteropathy (especially in non-food-responsive chronic inflammatory enteropathy) than in healthy controls [20], as well as in dogs presenting intestinal lymphangiectasia [9]. In the light of these published data, it is difficult to interpret our finding in healthy cheetahs; additionally, this is because a further study published on fecal proteomics similarly found this protein (more precisely, a transthyretin precursor) in the feces of healthy cats [7], leading to the consideration that this protein could be a normal finding in both healthy and diseased patients. It is possible to hypothesize that the (diagnostic) difference could be made from the amount found in the fecal substrate. Spots HC6 and HC8 correspond to the IgG Fc-binding protein (FCGBP), which is a Fc portion of the IgG molecule binding site present in the intestinal and colonic epithelia, where it binds monomeric and aggregated IgG, even if it is different from the known IgG Fc receptors [21]. Interestingly, in cheetahs, FCGBP was previously found in the protein profile of fecal samples of female cheetahs [18]. It is expressed in the mucus granule of the goblet cells in the intestine and in many other mucosal surfaces, and it is considered to have an important role in mucosal immunological defenses [22,23,24]. It was found to be increased in the intestine of human patients affected by ulcerative colitis and Crohn’s disease [25] and to be more abundant in pregnant polar bears’ feces [19]. The protein was also found in dogs suffering from lymphangiectasia and in healthy cats [7,9]; on the contrary, it was not described in healthy dogs and in dogs with chronic enteropathy [7,20]. Unfortunately, the few and hardly matching data available in the literature did not allow for an in-depth interpretation of our findings. Spots HC9 and DC9 were assigned to a 51.8 kDa polypeptide derived from the protein titin. This is a high molecular weight protein (381.6 kDa) that is also known as connectin and forms the intrasarcomeric filament [26]. Titin is a muscular structural protein that has been found to be increased in dogs affected by muscular dystrophy [27]; in this case, easier than in previous ones, it is reasonable to hypothesize that the source of that protein, and the reason for its presence in both the HC and DC groups, is very likely related to the high protein feeding habits of the subjects enrolled in this study. Similarly to transthyretin and FCGBP, titin was also found in the study about the protein profile stool samples of female cheetahs collected to evaluate the pregnancy status [18]. However, it remains difficult to justify the different quantities of titin found in the two groups. Spots HC12 and HC16 were identified as an 18 kDa polypeptide derived from distonin and an 833.7 kDa protein that is present in distinct isoforms; some of them are expressed in neural and muscle tissue, and others are expressed in the epithelial tissue and are essential for maintaining neuronal cytoskeleton integrity [28]. It is a multifunctional cytoskeletal linker protein, owing to the family of spectraplakins, expressed in various tissues with the role of keeping together the cytoskeleton and connecting it to the cellular junctions [29]. Spot HC13 was instead attributed to isopentenyl-diphosphate delta-isomerase 1, a protein previously found in dogs suffering from food-responsive diarrhea but which is interpreted as a presumable contaminant [8]. Not much information is available on this enzyme, and the few pieces of information that are available are difficult to correlate; for example, this enzyme is known to be able to alleviate plant drought stress [30], as well as to be involved in the cellular oxidative processes [31]. Considering the sodium/potassium-transporting ATPase subunit alpha-1 (spot HC14), very few data useful for a correlation with its presence in HC feces are present in the literature. A similar subunit has, for example, been found to be present in the urine of human patients diagnosed with immunoglobulin A nephropathy [32]. Finally, the last protein identified in healthy cheetahs, which is always hardly justifiable, was the protein disulfide-isomerase A6 (spot HC15). This protein is a folding catalyst (foldase) present in different tissues that is able to form and rearrange protein disulfide bonds; it has different functions and has been, for example, correlated with different cancers in men with a demonstrated potential for chemoresistance [33] as well as with patent ductus arteriosus [34].

Finally, in the feces of healthy and diseased cheetahs, eight further spots were found; they were supposed to correspond to five proteins (Chymotrypsin-C-like, Elastase-3B, Immunoglobulin kappa light chain, Immunoglobulin λ − 1 light chain, Immunoglobulin λ-light chain VLJ) that are also constantly present in the feces of healthy and diseased dogs, as shown in previous works [7,9]. This consideration can be reasonably assumed on account of contemplating the difficulties in comparing different investigations performed in different studies and on account the fact that they were however performed with the same methodology.

The main limitations of the present study are: (i) the possibility of comparing our results with the very few previous investigations already present in the literature [7,8,9,20], only one of which concerns felids (i.e., cats) [7]; and (ii) the low case load reported herein. Which species is being described must be considered, as well as how difficult it could be to include such patients. Additionally, the limitations associated with a routine clinic-pathological evaluation must be considered. Nevertheless, we believe it represents an important approach to the study of the fecal proteome in cheetahs, with a possible direct impact on the deepening of the knowledge of cheetahs’ GI physiopathology and of the discovery of clinical diagnostic/monitoring markers. It must be further stressed that the above discussion should be interpreted in light of the fact that very few data are available in the literature to draw definitive conclusions on the species investigated, but it represents a fundamental starting point for future studies and comparisons.

## 5. Conclusions

Proteomics has, among its main objectives, the objective of finding new clinical markers to be used for diagnostic and monitoring purposes in different physio-pathological contexts/substrates—such as feces, in our case. The two objectives of the present study are: (i) to investigate the fecal proteome in healthy cheetahs in order to provide a set of information on that environment, which is to be used for comparisons in future studies on diseased patients; (ii) to evaluate the main differences in patients suffering from GI disorders, finding, in our case, the presence of albumin only in the diseased patients. Future studies are needed to confirm and develop the present results.

## Figures and Tables

**Table 1:** Identification of proteins in healthy (HC) and diseased (DC) cheetahs.

Spot ID HC ^a^	Spot ID DC ^a^	Mr (kDa)/pI ^b^	Normalized Quantity (×10^3^) ^b^	Protein Name ^c^	Mr (kDa)/pI ^c^	Score ^c^	Sequence ^c^
HC1	-	135.5/9.3	175 ± 147	Collagen alpha-1 (II) chain	141.8/6.6	40	*GIAGPQGPR*
HC2	-	44.2/6.9	128 ± 50	Transthyretin	15.7/5.9	109	*GSAPAANVGVK*
HC3	-	40.7/6.4	307 ± 2	Transthyretin	15.7/5.9	144	*GSAPAANVGVK*
HC5	-	39.1/5.9	324 ± 48	Transthyretin	15.2/5.9	59	*KAADDTWEPFASG*
HC6	-	48.1/5.7	149 ± 27	IgG Fc-binding protein	571.6/5.1	128	*VLVENEHRG*
HC7 ^(1)^	DC7 ^(2)^	36.9/4.7 ^(1)^ 39.8/4.7 ^(2)^	401 ± 25 ^(1)^ 3298 ± 518 ^(2)^	n.d.	n.d.	n.d.	n.d.
HC8	-	35.7/4.4	460 ± 15	IgG Fc-binding protein	571.6/5.1	50	*LDSLVAQQLQSK*
HC9 ^(3)^	DC9 ^(4)^	51.8/4.5 ^(3)^ 50.2/4.4 ^(4)^	288 ± 130 ^(1)^ 7634 ^(2)^	Titin	3813.0/6.0	85	*APTPSPVR*
HC12	-	18.6/7.1	207 ± 40	Dystonin	833.7/5.2	73	*AVTTALK*
HC13	-	18.7/5.5	163 ± 36	Isopentenyl-diphosphate Delta-isomerase 1	26.4/5.6	55	*AANGEIK*
HC14	-	17.8/5.3	144 ± 43	Sodium/potassium-transporting ATPase subunit alpha-1	112.6/5.6	81	*CRGAGIKV*
HC15	-	16.4/4.8	125 ± 10	Protein disulfide-isomerase A6	48.1/4.9	51	*KAATALKD*
HC16	-	18.1/4.7	192 ± 6	Dystonin	833.7/5.2	83	*AVTTALK*
-	DC17	61.7/4.6	6477 ± 3214	Albumin	68.6/5.5	190	*KAPVSTPTLVEV*
-	DC18	62.6/5,5	6290 ± 864	n.d.	n.d.	n.d.	n.d.
-	DC19	57.3/4.7	5880 ± 1240	n.d.	n.d.	n.d.	n.d.
-	DC 20	58.9/5.7	7922 ± 922	Albumin	68.6/5.5	421	*KAPVSTPTLVEV*

^a^ Assigned spot ID as indicated in Figure 1 (1A: healthy cheetahs; 1B: diseased cheetahs); ^b^ Experimental values calculated from the 2DE maps by the PDQuest software; ^c^ Data obtained from MASCOT results (SwissProt databases). Numbers ^(1)^, ^(2)^, ^(3)^, and ^(4)^ are to link the spot name with the mr(kDa)/pI.

## Data Availability

All relevant data are contained within the article.
